# Ankle-angle-dependent shear wave velocity of the Achilles tendon and triceps surae during passive dorsiflexion in runners with Achilles tendinopathy

**DOI:** 10.3389/fspor.2026.1842666

**Published:** 2026-08-05

**Authors:** Yasuhiro Kunita, Naoki Ikeda, Takuya Nishioka, Shota Yamaguchi, Ayumi Yoshikawa, Daiki Hajima, Naoko Yabuno, Yuta Hashimoto, Kengo Harato, Takayuki Inami

**Affiliations:** 1 Graduate School of Health Management (Major in Public Health, Sport and Health Sciences), Keio University, Kanagawa, Japan; 2 Institute of Physical Education, Keio University, Kanagawa, Japan; 3 Sports Medicine Research Center, Keio University, Kanagawa, Japan

**Keywords:** Achilles tendon, elasticity imaging techniques, running, tendinopathy, triceps surae muscle

## Abstract

**Purpose:**

This study aimed to determine whether runners with mid-portion Achilles tendinopathy (Achilles tendinopathy runners [ATR]) show altered ankle-angle-dependent mechanical behavior of the Achilles tendon and triceps surae muscle–tendon unit during passive dorsiflexion.

**Methods:**

Twelve ATR and 11 control runners (CON) participated in this study (ATR: age 43.9 ± 12.0 years, running distance 38.3 ± 22.3 km/week; CON: age 41.2 ± 13.7 years, running distance 48.2 ± 29.7 km/week). The participants underwent device-controlled passive ankle dorsiflexion (DF) from 30° of plantar flexion (PF) to 80% of maximal DF at 1°/s in the prone position with the knee extended. Shear wave velocity (SWV) (m/s) was measured in the medial gastrocnemius (MG), lateral gastrocnemius (LG), soleus (SOL), and Achilles tendon (AT). The primary analysis used repeated-measures analysis of variance with angle (30/25/20/15/10/5/0° PF) and region (MG/LG/SOL/AT) as within-subject factors and group as the between-subject factor. A secondary triceps surae-only model (MG/LG/SOL) included 5/10/15° DF because the AT could not be measured beyond neutral.

**Results:**

In the primary model, the region × angle × group interaction was significant, and AT SWV was lower in ATR than in CON at 10° PF (ATR: 6.97 ± 0.49 vs. CON: 8.70 ± 0.51 m/s), 5° PF (ATR: 7.97 ± 0.43 vs. CON: 9.31 ± 0.45 m/s), and 0° PF (ATR: 9.15 ± 0.57 vs. CON: 11.30 ± 0.60 m/s) (all *p* < 0.05). The MG and SOL SWV were lower in ATR at all PF angles, whereas the LG showed no differences. In the secondary model, the MG and SOL remained lower in ATR across 5–15° DF.

**Conclusion:**

The ATR group showed lower Achilles tendon SWV near neutral and consistently lower MG and SOL SWV across ankle angles. These findings suggest altered ankle-angle-dependent mechanical behavior of the Achilles tendon and MG–SOL components of the muscle–tendon unit in runners with Achilles tendinopathy.

## 1 Introduction

Running imposes substantial repetitive loads on the Achilles tendon (AT), a key elastic structure for energy storage and return during stance. High cumulative tendon loading may contribute to overuse injury when recovery is insufficient (1, 2). Achilles tendinopathy is one of the most common running-related injuries, accounting for approximately 10% of all specific running pathologies in a systematic review (3). Its persistent symptoms are associated with reduced physical function and healthcare utilization (4), highlighting the need to clarify AT's mechanical characteristics in runners.

Achilles tendinopathy is characterized by load-related pain and functional limitations and is often accompanied by structural tendon changes. Conventional ultrasonography commonly reveals increased tendon thickness, altered echogenicity, and neovascularization, which support diagnosis and may reflect tendon remodeling (5, 6). However, the structural findings do not fully explain these symptoms or functional deficits. Tendinopathy is also associated with the altered neuromuscular function of the triceps surae, including changes in motor output and activation strategies during loading tasks (7). In this context, these observations suggest that a complete characterization of Achilles tendinopathy in runners may require the evaluation of both tendon and muscle behavior under standardized conditions. This raises the question of whether tissue-level mechanical properties, rather than morphology alone, differ in symptomatic runners and whether such differences depend on the joint angle.

Ultrasound shear wave elastography (SWE) quantifies *in vivo* soft tissue mechanical behavior using shear wave velocity (SWV) or shear modulus as surrogates of stiffness (5, 8). In healthy individuals, SWE during passive dorsiflexion (DF) shows clear angle-dependent stiffness profiles of the triceps surae, including muscle-specific slack angles (9–11). For the AT, the stiffness increases with DF; however, most studies have assessed only discrete positions, typically at 0° (neutral) (12, 13). Our recent work addressed this by continuously profiling the AT and triceps surae stiffness across angles in healthy runners (14). In Achilles tendinopathy, SWE studies have reported altered tendon mechanical properties at resting ankle positions (6, 15, 16). These alterations are accompanied by structural features such as tendon thickening, altered echogenicity, and neovascularization (5, 6). Together, these findings suggest that pathological changes in Achilles tendinopathy can affect local shear wave propagation and tissue-level mechanical behavior.

Importantly, such mechanical differences may not be uniform across ankle angles. During passive DF, AT tension and strain are expected to increase as the ankle moves from plantar flexion (PF) toward neutral (10, 12, 14). Therefore, disease-related differences in tendon mechanical behavior may become more apparent near neutral, where the tendon is expected to be under greater tensile loading than in a more plantarflexed position. This angle-specific perspective is clinically relevant because AT loading during running and rehabilitation does not occur at a single resting ankle position. Although slow passive DF does not reproduce running, it provides a standardized condition for evaluating tissue-level mechanical behavior while minimizing the confounding effects of voluntary muscle activation. Passive SWE may therefore help identify whether the symptomatic muscle–tendon unit shows altered stiffness profiles across ankle angles and may provide a biomechanical basis for considering ankle angle during load management in progressive rehabilitation.

Accordingly, this study aimed to determine whether runners with mid-portion Achilles tendinopathy show altered ankle-angle-dependent mechanical behavior of the AT and triceps surae muscle–tendon unit during passive DF. We hypothesized that runners with Achilles tendinopathy would show lower AT SWV as the ankle approached neutral because pathological changes in the tendon may become more evident when passive tendon tension increases during DF.

## 2 Methods

### 2.1 Study design

This cross-sectional study compared runners with mid-portion Achilles tendinopathy (Achilles tendinopathy runner [ATR]) and healthy control runners (CON). This study was conducted at the Institute of Physical Education, Keio University (Kanagawa, Japan) from August 19, 2025, to January 19, 2026. Sample size was calculated using the G*Power software (version 3.1.9.6; Düsseldorf, Germany). A pre-study statistical power analysis was performed for a three-factor repeated-measures analysis of variance with a within–between interaction using G*Power software (version 3.1.9.6; Düsseldorf, Germany). The model included group as the between-subject factor and region and ankle angle as within-subject factors. Because no previous study had examined continuous angle-dependent SWE profiles of the Achilles tendon and triceps surae in runners with Achilles tendinopathy using the same protocol, the expected effect size was set at *f* = 0.25 based on Cohen's convention for a medium effect. With *α* = 0.05 and power = 0.80, the analysis indicated that a minimum total sample size of 12 participants was required (17, 18).

### 2.2 Ethics approval and participants

This study was approved by the Research Ethics Committee of Keio University (approval no. 24-013) and adhered to the Declaration of Helsinki and relevant ethical guidelines. All participants provided written informed consent before participation.

The participants were recreational runners aged 18–60 years. A runner was defined as an individual who ran ≥ 15 km per week. Participants were instructed not to exercise on the day of testing. Twelve (9 men and 3 women) and 11 participants (8 men and 3 women) were included in the ATR and CON groups, respectively. Participant characteristics are summarized in Table 1. There were no between-group differences in age, height, body mass, weekly running distance, or time since the last running session.

**Table 1:** Characteristics of the study population.

Demographic	ATR (*n* = 12)	CON (*n* = 11)
Sex (male/female)	9/3	8/3
Age (y)	43.9 ± 12.0	41.2 ± 13.7
Height (cm)	169.3 ± 7.8	165.7 ± 9.0
Weight (kg)	60.4 ± 9.0	57.2 ± 8.6
Maximum dorsiflexion ROM (°)	30.9 ± 6.7	29.6 ± 3.4
Running distance per week (km)	38.3 ± 22.3	48.2 ± 29.7
Time since last running session (days)	1.8 ± 1.7	2.3 ± 2.1

Values are presented as means ± SDs.

Participants were eligible for the CON group if they (i) met the runner definition; (ii) had no current pain, tenderness, or inflammatory symptoms in the AT or calf; and (iii) had no history of major lower limb trauma or surgery, including AT injury, that could affect ankle or foot function. Participants were eligible for the ATR group if they (i) met the runner definition, (ii) reported AT pain or discomfort persisting for ≥ 3 months, (iii) had pain localized to the mid-portion of the AT (2–6 cm proximal to the calcaneal insertion) that was aggravated by activity and/or at rest, (iv) had been diagnosed with Achilles tendinopathy by a physician, and (v) had a Victorian Institute of Sports Assessment–Achilles (VISA-A) score ≤ 80. The diagnosis of Achilles tendinopathy was based on these clinical criteria and a previous physician diagnosis. Ultrasonographic findings, including tendon thickness, hypoechogenicity, fibrillar pattern irregularity, and neovascularization, were recorded only to characterize tendon structural features in the Achilles tendinopathy group. Symptom duration was recorded in the ATR group, and symptom severity was assessed using the VISA-A. Participants were excluded if they had insertional Achilles tendinopathy, history of AT rupture, any other major foot or ankle pathology, or surgery unrelated to the AT that could affect function. Additional exclusion criteria included neurological, cardiovascular, or systemic diseases that could influence exercise performance (e.g., diabetes mellitus), pregnancy or breastfeeding, and long-term use of topical or oral corticosteroids. In the ATR group, the analyzed limb was the symptomatic side. In the CON group, the analyzed limb was the dominant leg, defined as the self-reported preferred leg for kicking. In the CON group, the analyzed limb was the dominant leg, defined as the self-reported preferred leg for kicking. This definition was consistent with a previous biomechanical study of running-related limb dominance (19).

In the ATR group, tendon structural features were assessed using ultrasonography as a clinical descriptor of tendon pathology. Tendon thickness was measured, and hypoechogenicity, fibrillar pattern irregularity, and neovascularization were recorded as present or absent (yes/no).

### 2.3 Experimental setup and passive dorsiflexion procedure

**Figure 1:**
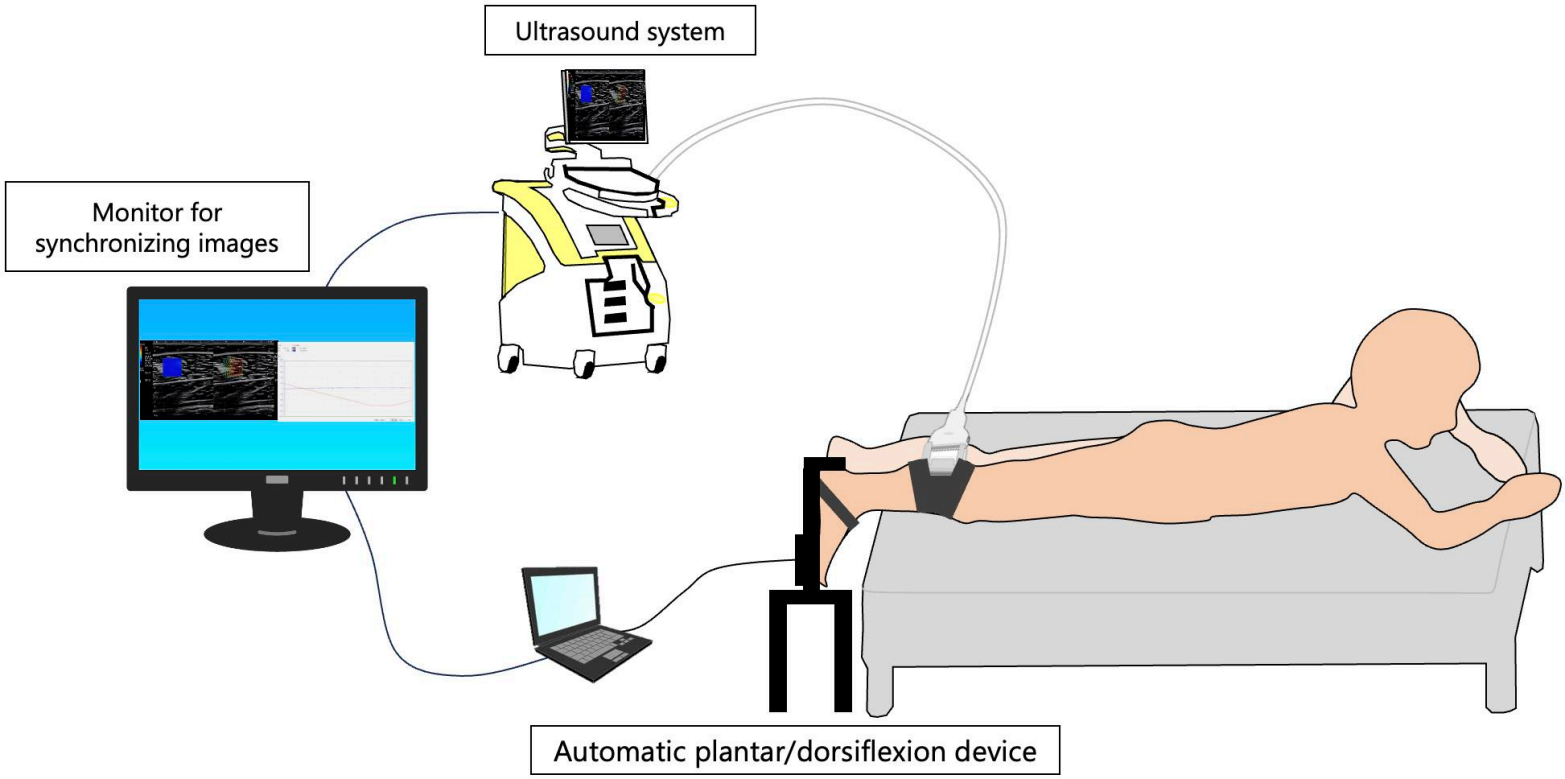
Experimental setup and passive dorsiflexion procedure. The participants were placed in the prone position on a dynamometer attachment, with the dominant foot secured by a belt. Ultrasound equipment was used to perform shear wave elastography (SWE) of the triceps surae and Achilles tendon (AT). For MG, LG, and SOL measurements, the ultrasound probe was fixed to the lower leg using a soft pad and an elastic band to minimize movement and avoid probe pressure. For the AT measurements, the probe was held manually because of interference with the dynamometer attachment. The dynamometer and ultrasound system were connected to a single display to synchronize shear wave velocity (SWV) with the joint angle.

The experimental setup is shown in Figure 1. The experimental setup and passive DF protocol were based on previously established procedures (14). During passive dorsiflexion testing, participants were asked to remain relaxed and to report any Achilles tendon pain experienced during the procedure. Each participant lay prone on a bed with the hip in a neutral position and the knee fully extended (0°). Passive ankle DF was performed using an automatic PF/DF device (S-18129; Takei Scientific Instruments Co., Ltd., Japan). Each foot was secured to a footplate with a belt, and the rotation axis of the dynamometer was aligned with the medial and lateral malleoli. The torque and ankle joint angle data were acquired directly from the output system of the dynamometer. These signals were recorded at 1000 Hz and collected on a personal computer via a relay device (AIO AI-1608AY-USB; Takei Scientific Instruments Co., Ltd., Japan) using a dedicated software (version 1.1.0 [S-19040]). First, the maximum passive DF angle was determined. The ankle was passively dorsiflexed at 1°/s, and the participants pressed a handheld switch when they reached their maximum tolerable stretch on the posterior lower leg. An angle corresponding to 80% of the individual maximum DF angle was calculated to reduce the likelihood of increased plantar-flexor activation near the end range during passive DF (14, 20). For elastography, passive DF was performed from 30° PF to 80% of the maximum DF angle at 1°/s.

During passive DF, the SWV (m/s) of the medial gastrocnemius (MG), lateral gastrocnemius (LG), soleus (SOL), and AT were measured using SWE. For statistical analysis, the SWV was extracted at 30° PF, 25° PF, 20° PF, 15° PF, 10° PF, 5° PF, and 0° (neutral) and at 5° DF, 10° DF, and 15° DF for the triceps surae-only model.

### 2.4 Shear wave elastography measurement

An ultrasound system with SWE capability (Aplio a Verifia, Canon Medical Systems Corporation, Japan) and a linear probe (65 mm, 8–14 MHz) were used to acquire B-mode ultrasound images in the longitudinal plane with color mapping of the SWV for the triceps surae and AT during passive DF. All measurements were performed by a licensed physical therapist with 13 years of clinical experience and extensive expertise in musculoskeletal ultrasonography. The probe was positioned along the longitudinal axis of each muscle or tendon and perpendicular to the skin. Alignment was confirmed by visualization of both the superficial and deep aponeuroses on B-mode imaging. For muscle measurements, the probe orientation was adjusted to maximize fascicle visibility and align the imaging plane as parallel as possible to the dominant fiber direction to mitigate anisotropy-related bias in SWE measurements (21). The probe pressure was minimized. The smoothing level was set at an intermediate spatial level (3/5). To standardize the probe positioning, skin markings were made with the lower leg in a resting position at the edge of the bed. The measurement sites were marked as follows: the maximal bulge of the MG and LG at approximately 30% of the proximal lower leg length, the SOL distal to the myotendinous junction of the LG (22), and the AT at the site of the greatest tendon thickness, approximately 2 cm from its insertion. The probe placement locations were marked on the skin to maintain probe-position consistency during each participant's measurements.

For the MG, LG, and SOL, the probe was mounted on a custom-made soft pad and secured to the lower leg using an elastic band to prevent probe movement while avoiding excessive compression. In contrast, the AT could not be measured using this fixation method because of interference with the dynamometer attachment. Therefore, the operator manually held the probe in minimal contact to avoid compression. A gel pad was placed over the concave tendon surface, and the probe was positioned on top of the gel pad during the AT measurements. Proper alignment was verified by clear visualization of the tendon boundaries and stable shear wave propagation.

To account for muscle conditioning, five cycles of passive ankle movement were performed at 5°/s from 30°PF, where passive torque is considered minimal in a slack region, to 80% of the maximum DF angle (14, 20). Subsequently, SWE measurements were obtained at each site (MG, LG, SOL, and AT) during passive DF at 1°/s, with a 1-min rest period between the sites. The measurement order was fixed across participants as follows: MG, LG, SOL, and AT. Finally, to check for potential stretching effects from repeated measurements, the SWE of the MG was repeated at the end of the protocol.

Each site was measured thrice. Based on a prior validation of this SWE protocol using the same experimental setup, acquisition procedures, and analysis workflow (14), one representative measurement per site was used for analysis in the present study. If the participant moved during acquisition or if the SWE image was visibly disturbed, the second or third acquisition was selected based on predefined image-quality criteria. The image-quality criteria included clear visualization of the target tissue in the longitudinal plane, stable SWE color mapping, absence of visible artifacts in the region of interest, and maintenance of probe alignment during passive dorsiflexion. Based on a prior validation of this SWE protocol using the same experimental setup, acquisition procedures, and analysis workflow, intraclass correlation coefficients [ICC (3,1), absolute agreement] and coefficients of variation (CV) were calculated between the three repeated measurements and the subsequent single measurement in six participants to evaluate the reliability of using a single representative measurement. The ICC values were high across all sites (ICC = 0.85–0.99), and the CVs were low (CV = 2.4%–7.7%), indicating excellent intra-rater measurement reliability (14). The total experiment duration, including preparation and data collection, was approximately 90 min per participant.

### 2.5 Statistical analyses

Statistical significance was set at *p* < 0.05. Analyses were performed using SPSS (version 30.0; IBM, Armonk, NY, USA). Continuous data are reported as means ± standard deviations unless otherwise indicated. Between-group differences in participant characteristics were examined using independent-samples t-tests. To examine the presence of a stretching effect (i.e., the influence of repeated passive DF on stiffness), the SWV at 80% of the maximum DF angle was compared between the first and second MG measurements (MG-1st vs. MG-2nd). The normality of the paired differences was assessed using the Shapiro–Wilk test. If normality was satisfied, a paired t-test was used to test for systematic differences between MG-1st and MG-2nd. Subsequently, SWV was analyzed using repeated-measures analysis of variance models with Greenhouse–Geisser corrections applied when sphericity was violated. In the primary model, the angle (30° PF, 25° PF, 20° PF, 15° PF, 10° PF, 5° PF, and 0° [neutral]) and region (MG/LG/SOL/AT) were within-subject factors, whereas group (ATR vs. CON) was a between-subject factor. In the secondary model, only the triceps surae (MG/LG/SOL) was analyzed across an extended angle range, including DF (30° PF to 15° DF), because the AT could not be measured beyond 0° owing to device constraints. *Post-hoc* comparisons were performed using the Bonferroni adjustment. Effect sizes were reported as partial eta squared (*η*p^2^). Between-group differences in SWV were additionally summarized using mean differences and 95% confidence intervals where applicable.

## 3 Results

No participant reported Achilles tendon pain during passive dorsiflexion testing. here was no significant difference between the first and second MG measurements (MG-1st vs. MG-2nd) at 80% of the maximum DF angle (5.63 ± 1.14 m/s vs. 5.57 ± 1.14 m/s, *p* = 0.38), indicating no apparent effect of repeated passive DF on stiffness.

### 3.1 Clinical characteristics of the Achilles tendinopathy runner group

The clinical characteristics of the ATR group are summarized in Table 2. The duration of symptoms was 48.3 ± 79.9 months, and the VISA-A score was 54.0 ± 18.5. Ultrasound structural findings revealed a tendon thickness of 7.4 ± 2.0 mm. Hypoechogenicity and fibrillar pattern irregularities were present in 9/12 participants, and neovascularization was present in 6/12 participants.

**Table 2:** Clinical characteristics of the ATR group.

Demographic	Value
Symptom duration (months)	48.3 ± 79.9
VISA-A (points)	54.0 ± 18.5
Tendon thickness (mm)	7.4 ± 2.0
Hypoechogenicity (yes/no)	9/12
Fibrillar pattern irregularity (yes/no)	9/12
Neovascularization (yes/no)	6/12

Values are presented as means ± SDs, unless otherwise indicated.

ATR, Achilles tendinopathy runner; VISA-A, Victorian Institute of Sport Assessment Achilles.

### 3.2 Angle-dependent shear wave velocity profiles and between-group differences

**Figure 2:**
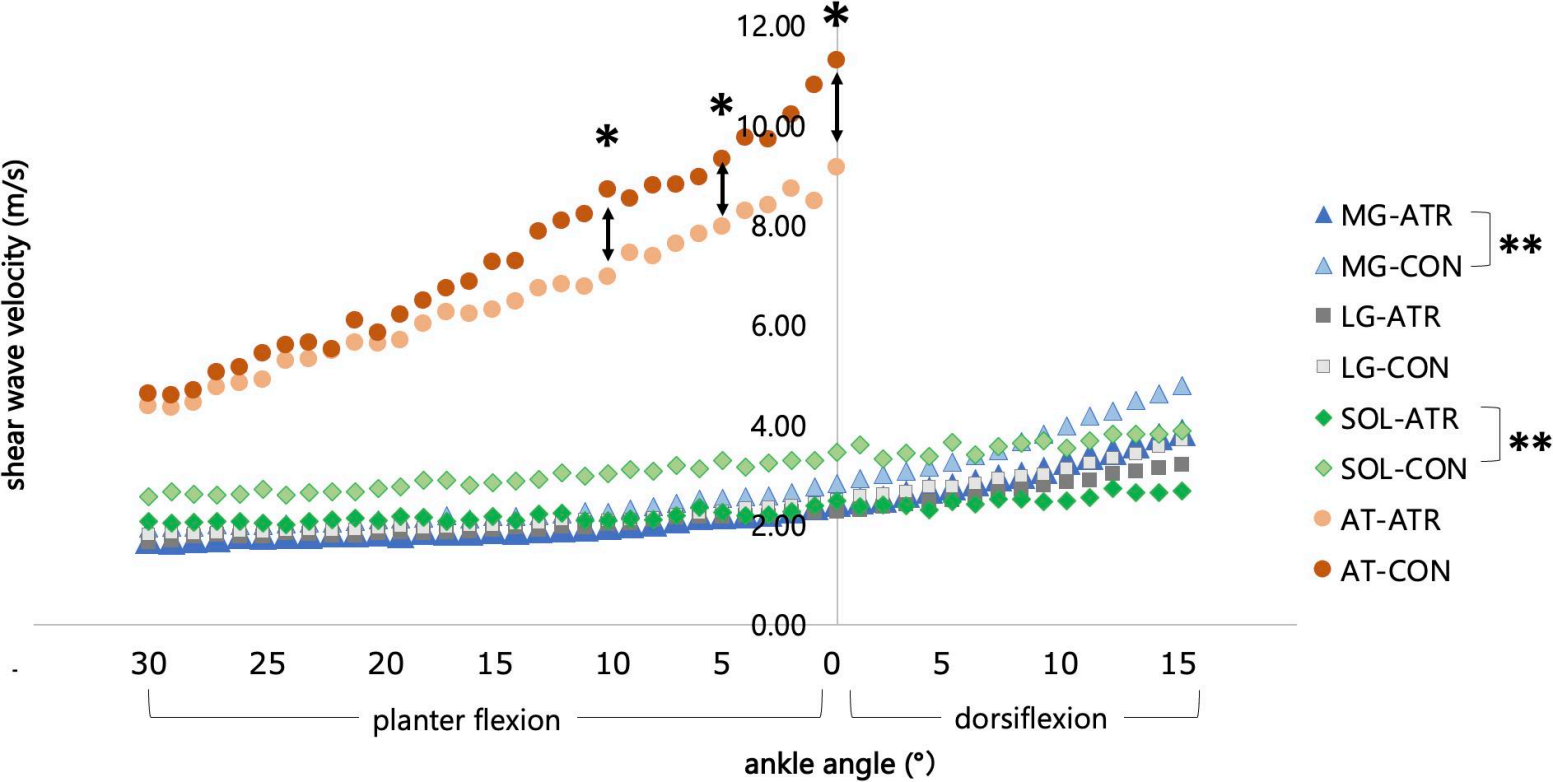
Angle-dependent shear wave velocity profiles of the triceps surae and Achilles tendon across plantarflexion and dorsiflexion. Mean shear wave velocitiy (SWV, m/s) of the medial gastrocnemius (MG), lateral gastrocnemius (LG), soleus (SOL), and Achilles tendon (AT) during device-controlled passive ankle dorsiflexion in runners with Achilles tendinopathy (Achilles tendinopathy runner [ATR]) and control runners (CON). Ankle angles are expressed as plantar flexion (PF) from 30° to 0° (0° = neutral) and dorsiflexion (DF) from 5° to DF15°. The MG, LG, and SOL were assessed across the full range (PF30°–DF15°), whereas AT measurements were available only up to 0°, owing to device limitations. Values are presented as means ± SDs. Results of repeated-measures ANOVA (*p* < 0.05), followed by Bonferroni's *post-hoc* test. *Significant difference between the ATR and CON groups in the AT. **There were significant differences between groups within the same muscle at all angles.

The descriptive values of the SWV and derived shear modulus at each ankle angle are listed in Table 3. Between-group differences in SWV with 95% confidence intervals for the primary model are presented in Supplementary Table S1. Figure 2 shows the overall angle-dependent SWV profiles for the MG, LG, SOL, and AT in both groups.

**Table 3:** Shear wave velocity and shear modulus at each ankle joint angle.

Group	Region	Measure	30° PF	25° PF	20° PF	15° PF	10° PF	5° PF	0°	5° DF	10° DF	15° DF
ATR	MG	SWV	1.72 ± 0.28	1.78 ± 0.22	1.86 ± 0.24	1.92 ± 0.24	1.99 ± 0.24	2.20 ± 0.26	2.47 ± 0.27	2.76 ± 0.36	3.30 ± 0.44	3.90 ± 0.64
		SM	3.01 ± 1.04	3.21 ± 0.83	3.52 ± 0.87	3.74 ± 0.89	3.99 ± 0.90	4.88 ± 1.09	6.15 ± 1.27	7.75 ± 1.93	11.09 ± 2.89	15.60 ± 4.95
	LG	SWV	1.69 ± 0.27	1.81 ± 0.27	1.88 ± 0.25	1.93 ± 0.27	2.04 ± 0.26	2.19 ± 0.29	2.31 ± 0.18	2.55 ± 0.27	2.91 ± 0.35	3.28 ± 0.50
		SM	2.91 ± 1.02	3.35 ± 1.00	3.58 ± 0.94	3.79 ± 1.03	4.24 ± 1.09	4.87 ± 1.32	5.35 ± 0.79	6.59 ± 1.36	8.60 ± 2.02	10.98 ± 3.27
	SOL	SWV	2.13 ± 0.43	2.19 ± 0.64	2.22 ± 0.71	2.32 ± 0.82	2.21 ± 0.73	2.51 ± 1.02	2.73 ± 1.06	2.64 ± 1.15	2.59 ± 0.93	2.80 ± 0.97
		SM	4.69 ± 2.16	5.15 ± 3.29	5.38 ± 3.64	5.99 ± 4.70	5.39 ± 4.00	7.24 ± 6.81	8.47 ± 7.73	8.19 ± 8.27	7.51 ± 5.99	8.70 ± 6.80
	AT	SWV	4.30 ± 0.46	4.80 ± 0.81	5.55 ± 1.29	6.20 ± 1.11	6.96 ± 1.20	7.93 ± 1.18	9.24 ± 1.89	—	—	—
		SM	18.69 ± 3.88	23.61 ± 8.28	32.34 ± 14.50	39.60 ± 13.92	49.70 ± 16.96	64.23 ± 18.86	88.65 ± 37.48	—	—	—
CON	MG	SWV	1.95 ± 0.20	2.01 ± 0.19	2.13 ± 0.21	2.19 ± 0.19	2.28 ± 0.18	2.56 ± 0.23	2.85 ± 0.28	3.32 ± 0.35	4.03 ± 0.46	4.84 ± 0.56
		SM	3.85 ± 0.79	4.07 ± 0.76	4.58 ± 0.90	4.83 ± 0.85	5.24 ± 0.83	6.58 ± 1.18	8.21 ± 1.59	11.11 ± 2.32	16.44 ± 3.77	23.72 ± 5.43
	LG	SWV	1.81 ± 0.20	1.87 ± 0.23	1.95 ± 0.23	2.01 ± 0.22	2.12 ± 0.21	2.29 ± 0.23	2.49 ± 0.27	2.76 ± 0.29	3.10 ± 0.37	3.69 ± 0.55
		SM	3.27 ± 0.72	3.61 ± 0.92	3.94 ± 0.91	4.10 ± 0.90	4.56 ± 0.90	5.31 ± 1.08	6.30 ± 1.28	7.68 ± 1.61	10.01 ± 2.46	13.56 ± 4.22
	SOL	SWV	2.59 ± 0.36	2.75 ± 0.41	2.78 ± 0.51	2.91 ± 0.63	3.08 ± 0.56	3.34 ± 0.65	3.49 ± 0.81	3.68 ± 0.86	3.56 ± 0.69	3.88 ± 0.74
		SM	6.79 ± 1.91	7.71 ± 2.32	7.94 ± 2.90	8.84 ± 3.65	10.00 ± 3.42	12.01 ± 4.58	12.96 ± 6.05	14.36 ± 7.22	12.36 ± 5.44	15.24 ± 5.62
	AT	SWV	4.59 ± 0.43	5.39 ± 0.75	5.80 ± 0.99	7.16 ± 1.07	8.68 ± 1.52	9.38 ± 1.49	11.17 ± 2.29	—	—	—
		SM	21.23 ± 4.04	29.68 ± 8.00	34.46 ± 11.37	52.46 ± 15.97	78.01 ± 25.14	90.11 ± 29.62	129.44 ± 50.80	—	—	—

Values are mean ± SD. 0 = neutral. AT is unavailable beyond 0°.

ATR, Achilles tendinopathy runnner; CON, control; MG, medial gastrocnemius; LG, lateral gastrocnemius; SOL, soleus; AT, Achilles tendon; PF, planter flexion; DF, dorsi flexion; SWV, shear wave velocity (m/s); SM, shear modulus (kPa).

For the primary model (region: MG/LG/SOL/AT, angle: 30° PF to 0° [neutral]), Mauchly's test indicated violations of sphericity; therefore, Greenhouse–Geisser corrections were applied. A significant region × angle × group interaction was observed (Table 4), indicating that between-group differences in SWV depended on both the region and ankle angle.

**Table 4:** Repeated-measures ANOVA results for SWV (GG-corrected).

Effect	Model 1 df (GG)	Model 1 df error (GG)	Model 1 F	Model 1 p	Model 1 ηp^2^
Region	1.549	32.534	370.338	<0.001	0.946
Region × Group	1.549	32.534	2.883	0.082	0.121
Angle	1.974	41.460	159.297	<0.001	0.884
Angle × Group	1.974	41.460	5.141	0.010	0.197
Region × Angle	2.459	51.629	88.151	<0.001	0.808
Region × Angle × Group	2.459	51.629	3.251	0.037	0.134

Effect	Model 2 df (GG)	Model 2 df error (GG)	Model 2 F	Model 2 p	Model 2 ηp^2^
Region	1.174	24.652	6.742	0.012	0.243
Region × Group	1.174	24.652	4.979	0.030	0.192
Angle	2.124	44.595	241.368	<0.001	0.920
Angle × Group	2.124	44.595	6.573	0.003	0.238
Region × Angle	4.106	86.235	24.423	<0.001	0.538
Region × Angle × Group	4.106	86.235	1.089	0.368	0.049

Model 1: MG/LG/SOL/AT across 30° PF – 0°.

Model 2: MG/LG/SOL across 30° PF – 15° DF, df are GG- adjusted.

GG, Greenhouse-Geissar; MG, medial gastrocnemius; LG, lateral gastrocnemius; SOL, soleus; AT, Achilles tendon; PF, planter flexion; DF, dorsi flexion.

**Figure 3:**
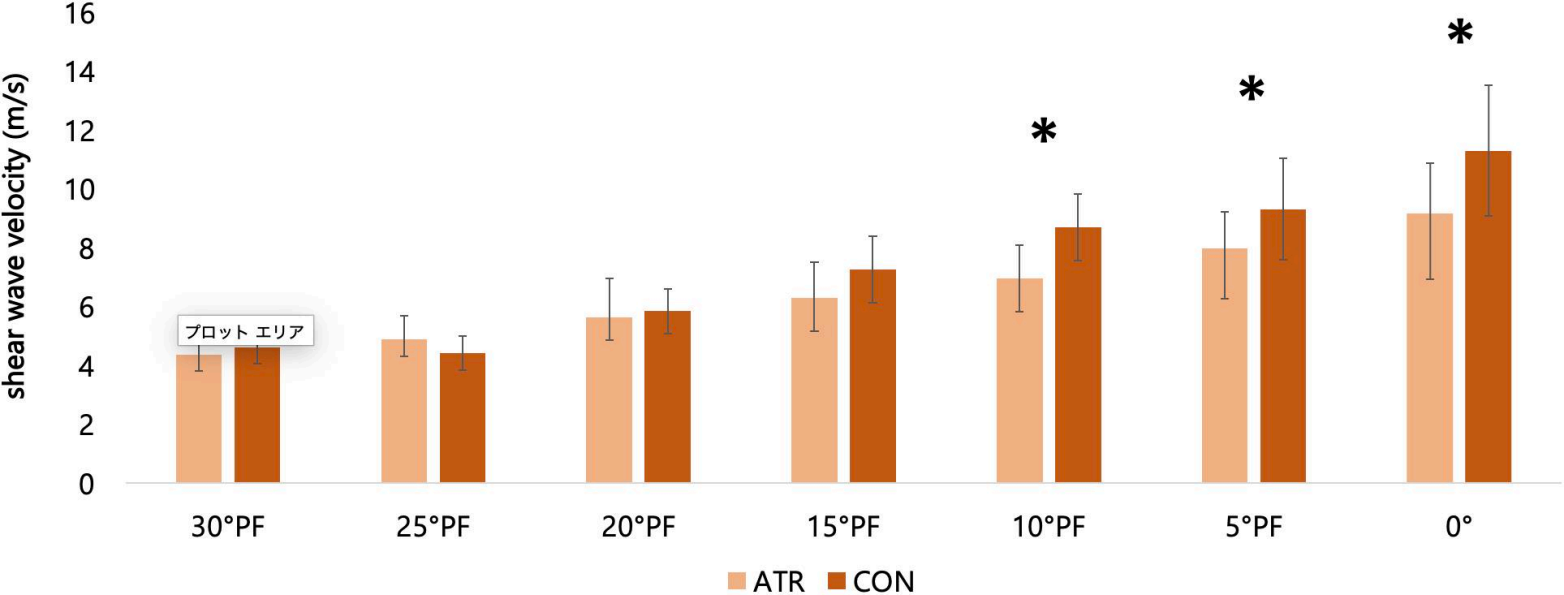
Between-group differences in Achilles tendon shear wave velocity. Achilles tendon (AT) shear wave velocity (SWV, m/s) in runners with Achilles tendinopathy (Achilles tendinopathy runner [ATR]) and control (CON) runners at 30–0° plantar flexion (PF). AT SWV was lower in the ATR group than in the CON group at PF10°, PF5°, and 0° (*p* < 0.05; see Table 4). Values are presented as means ± SDs. *Significant difference between the ATR and CON groups.

Post-hoc comparisons with Bonferroni adjustment showed that AT SWV was lower in ATR than in CON at 10° PF, 5° PF, and 0° (neutral) (*p* < 0.05), whereas MG and SOL SWV were lower in ATR across all PF angles (all Bonferroni-adjusted *p* < 0.05). The LG showed no significant between-group differences across PF angles. Figure 3 highlights the between-group differences in AT SWV at 30° PF to 0° in 5° increments.

For the secondary model (triceps surae only) (region: MG/LG/SOL, angle: 30° PF to 15° DF), AT data were not available beyond 0° owing to device limitations. In this model, region × angle × group interactions were not significant, whereas region × group and angle × group interactions were significant (Table 4). *Post-hoc* comparisons with Bonferroni adjustment showed that the MG and SOL SWV remained lower in the ATR group across the dorsiflexed range (5° DF to 15° DF) (*p* < 0.05), whereas the LG showed no significant between-group differences across most angles; a borderline difference was observed at 15° DF (Bonferroni-adjusted *p* = 0.050).

## 4 Discussion

This study characterized the angle-dependent stiffness profiles of the triceps surae and AT using SWE in the ATR group compared with the CON group. The primary model (MG/LG/SOL/AT across 30° PF to 0° [neutral]) showed a significant region × angle × group interaction, indicating that group differences in SWV were dependent on both the region and ankle angle. *Post-hoc* comparisons showed that the AT SWV was lower in the ATR group than in the CON group as the ankle approached the neutral position, with significant between-group differences at 10° PF, 5° PF, and 0° (neutral), whereas the MG and SOL SWV were consistently lower in the ATR group than in the CON group at all PF angles. In the secondary model that extended the range to DF for the triceps surae only, the MG and SOL remained lower in ATR across DF angles, whereas the LG showed minimal differences. The present data directly show that AT SWV was lower in the ATR group than in the CON group near neutral and that MG and SOL SWV were lower across the tested angles. These findings indicate altered ankle-angle-dependent SWV profiles under passive dorsiflexion. However, the underlying mechanisms cannot be determined from the present measurements alone because tendon composition, fascicular sliding, muscle activation, and tendon force were not directly assessed.

The progressive increase in SWV with DF and the clear muscle–tendon differences align with the known length and strain dependence of SWE-derived stiffness measures (9, 10). Our device-controlled passive DF at a constant velocity, together with standardized probe positioning and procedures to mitigate anisotropy, supports measurement consistency (21). This protocol matched that of our previous study on healthy runners (14), enabling the direct interpretation of angle-dependent profiles, although absolute SWV values remain system- and setting-dependent (12, 23). In this study, the AT SWV in the CON group at 0° (neutral) fell within previously reported ranges (12, 13, 24). The absolute MG and LG values at 0° and 15° DF were slightly lower than published estimates after converting shear modulus to SWV (11, 25), but the angle-dependent pattern was consistent with the previous reports. Thus, while direct agreement in absolute magnitude was limited, the similar change across ankle positions supports the overall validity of the present findings. By contrast, the SOL values fall within the range of previous studies, although considerable between-study variability has been reported, likely because of measurement depth and intramuscular heterogeneity (22, 26).

A key finding was that AT SWV was lower in the ATR group than in the CON group from 10° PF to 0° (neutral), whereas no differences were detected at more plantarflexed angles (30° PF to 15° PF). This angle-specific pattern suggests that disease-related differences in tendon mechanical behavior may become more apparent as the ankle angle approaches neutral position, where passive tension and tendon strain are expected to increase. Previous SWE studies have shown lower Achilles tendon stiffness in Achilles tendinopathy, but these observations were limited to maximal plantar flexion, 0°, or a single resting ankle position (6, 25). Our study extends this literature by showing that the reduction in Achilles tendon SWV becomes evident from 10° plantar flexion during passive dorsiflexion. This is a novel finding and highlights the importance of angle-specific assessment when detecting tendon abnormalities in Achilles tendinopathy. The ATR group showed greater tendon thickness and, in some runners, ultrasound features such as hypoechogenicity, fibrillar irregularity, and neovascularization, which are consistent with structural changes associated with Achilles tendinopathy. Tendinopathic tissues can show compositional and microstructural changes, such as increased water content, proteoglycan-related matrix changes, and collagen disorganization, which can alter shear wave propagation and thus affect the SWV measured *in vivo* (27). Accordingly, these structural and compositional abnormalities may contribute to the lower SWV observed in the ATR group, especially near the neutral ankle position. However, these abnormalities do not necessarily translate into reduced tensile properties (27). This difference is expected because the SWE-derived SWV reflects a local, modality-specific estimate of mechanical behavior influenced by hydration, matrix organization, and the local stress state, whereas tensile tests assess macroscopic tissue behavior under different boundary conditions. Therefore, our findings are best interpreted as an angle-dependent shift in *in vivo* tendon mechanical behavior near neutral rather than as evidence of reduced tensile strength.

Beyond the tendon, MG and SOL SWV were consistently lower in the ATR group than in the CON group across PF angles, and these differences persisted when DF angles were included in the triceps surae-only model. The lower MG and SOL SWV observed in the ATR group may reflect broader alterations in the muscle–tendon unit. Previous studies have reported reduced plantarflexor strength and endurance in runners with mid-portion Achilles tendinopathy, which may be explained, at least in part, by reduced SOL force-generating capacity (28). Achilles tendinopathy has also been associated with altered neuromuscular behavior, including higher electromyography activity during eccentric calf tasks and muscle-dependent changes in motor output (7, 29). In addition, reduced gastrocnemius dynamic stiffness measured by myotonometry has been reported in individuals with symptomatic Achilles tendons (30). These findings provide possible explanations for the lower MG and SOL SWV observed in the present study. However, because muscle activation, force-generating capacity, and tendon loading were not directly measured, neuromuscular adaptation and altered muscle–tendon load sharing should be interpreted as possible mechanisms rather than direct findings from the present data. Future studies should test these hypotheses under active loading conditions and longitudinal designs.

LG SWV showed no clear between-group differences across angles in either model, suggesting that passive LG stiffness was relatively preserved under our standardized passive dorsiflexion protocol. A possible explanation is that the Achilles tendon does not function as a single uniform structure, but is composed of distinct fascicular components arising from the MG, LG, and SOL, which twist as they merge distally. MG fascicles run relatively parallel, whereas LG and SOL fascicles show greater torsion, and this twist pattern has been reported to vary between individuals (31, 32). In addition, normal tendons deform non-uniformly during passive dorsiflexion, with greater displacement in the middle and deep portions than in the superficial portion, suggesting non-uniform load transfer and relative sliding within the tendon (33). On the other hand, in Achilles tendinopathy, intra-tendinous sliding has been reported to decrease, particularly in the superficial-to-middle region (34). Taken together, these anatomical and mechanical features may explain why LG SWV showed no clear between-group difference. However, the present study did not assess tendon fascicle-specific loading, intra-tendinous sliding, or regional tendon displacement. Therefore, the interpretation that MG- or SOL-related pathways may be more affected than the LG-related pathway should be considered hypothetical based on previous anatomical and imaging studies.

Clinically, the AT deficit that emerged from 10° PF to 0° (neutral) suggests that clinically relevant differences are not confined to DF positions, but may already be present in the late plantarflexed range. This may be important when considering running mechanics and rehabilitation progressions that move the ankle toward a neutral position under increasing tendon tension. The consistent reductions in MG and SOL SWV across angles further suggest that rehabilitation should address not only the tendon but also the calf muscle capacity and neuromuscular function. Exercise-based loading programs, including eccentric training and heavy slow resistance, have demonstrated clinical benefits in Achilles tendinopathy and have provided a pragmatic framework for progressive loading in runners (35, 36). However, the present passive SWE protocol does not directly simulate running or rehabilitation exercises. Therefore, the present findings should be interpreted as baseline biomechanical information for considering ankle angle during load management, and implications for progressive strengthening, graded tendon loading, or stretching should be tested in prospective intervention studies.

This study has some limitations. First, the plantar flexion range started at 30° PF because of the mechanical limits of the dynamometer device, whereas some previous protocols used more plantarflexed starting positions (e.g., 50° PF) (37). This may limit direct comparison of SWV profiles in the slackest region and earliest portion of the loading response. However, because Achilles tendon stiffness has been reported to increase in a largely linear manner across ankle dorsiflexion, the present findings still help define the mid-range behavior of the tendon. Together with prior data from more plantarflexed positions and future studies examining the dorsiflexed range, these results may contribute to a more complete understanding of Achilles tendon mechanical behavior across the full ankle range. Second, AT SWV could not be measured beyond 0° because ultrasound shear wave elastography has an upper measurement limit that was exceeded at more dorsiflexed positions. Therefore, tendon behavior during DF could not be evaluated directly. Even so, the present results identify the range up to neutral where between-group differences became evident and provide a basis for future studies extending into dorsiflexion. Third, although the final sample exceeded the minimum total sample size estimated in the power analysis, the sample size remained modest. Therefore, the study may have limited power to detect small between-group effects or to examine the influence of participant characteristics such as sex. In addition, the repeated-measures design involved multiple regions and ankle angles, which increased the number of statistical comparisons. Greenhouse–Geisser corrections and Bonferroni-adjusted *post-hoc* comparisons were applied to reduce the risk of inflated Type I error. Nevertheless, the possibility of residual Type I error due to multiple testing cannot be completely excluded. Fourth, the analyzed limb in the CON group was selected based on kicking dominance because the CON runners had no symptomatic limb. This criterion was chosen to standardize limb selection *a priori* and because kicking dominance has been used in previous studies of running-related limb asymmetry (19). However, kicking dominance may not fully reflect running-specific support or propulsion limb preference. Fifth, the examiner was not blinded to participant group allocation, which may have introduced measurement bias, although standardized probe positioning, device-controlled passive dorsiflexion, and image-quality criteria were used to reduce operator-dependent variability. Finally, the AT was assessed at the site of greatest tendon thickness within the mid-portion region. This approach allowed assessment of the most clinically relevant pathological region, but the exact measurement location may have differed between participants and may have influenced between-subject comparisons.

In conclusion, runners with mid-portion Achilles tendinopathy showed lower AT SWV during passive ankle DF as the ankle approached neutral position, with differences becoming evident from 10° PF to 0° (neutral) compared with control runners. The MG and SOL SWV were consistently lower across the PF and DF angles, whereas the LG showed minimal between-group differences. These findings indicate altered region-specific stiffness profiles of the calf muscle–tendon unit in Achilles tendinopathy and may support rehabilitation strategies that target both the AT and MG–SOL complex, with attention to the ankle angle.

## Data Availability

The raw data supporting the conclusions of this article will be made available by the authors, without undue reservation.
